# The clerodane diterpene casearin J induces apoptosis of T-ALL cells through SERCA inhibition, oxidative stress, and interference with Notch1 signaling

**DOI:** 10.1038/cddis.2015.413

**Published:** 2016-01-28

**Authors:** C De Ford, B Heidersdorf, F Haun, R Murillo, T Friedrich, C Borner, I Merfort

**Affiliations:** 1 Department of Pharmaceutical Biology and Biotechnology, Albert Ludwigs University Freiburg, Freiburg, Germany; 2 Spemann Graduate School of Biology and Medicine (SGBM), Albert Ludwigs University Freiburg, Freiburg, Germany; 3 Faculty of Chemistry and Pharmacy, Albert Ludwigs University Freiburg, Freiburg, Germany; 4 Institute of Molecular Medicine and Cell Research, Albert Ludwigs University Freiburg, Freiburg, Germany; 5 Escuela de Química and CIPRONA, Universidad de Costa Rica, San José, Costa Rica; 6 Institut für Biochemie, Albert Ludwigs University, Freiburg, Germany; 7 Centre for Biological Signaling Studies (BIOSS), Freiburg, Germany

## Abstract

T-cell acute lymphoblastic leukemia (T-ALL) is an aggressive hematologic malignancy that preferentially affects children and adolescents. Over 50% of human T-ALLs possess activating mutations of Notch1. The clerodane diterpene casearin J (CJ) is a natural product that inhibits the sarcoendoplasmatic reticulum calcium ATPase (SERCA) pump and induces cell death in leukemia cells, but the molecular mechanism of cytotoxicity remains poorly understood. Here we show that owing to SERCA pump inhibition, CJ induces depletion of the endoplasmic reticulum calcium pools, oxidative stress, and apoptosis via the intrinsic signaling pathway. Moreover, Notch1 signaling is reduced in T-ALL cells with auto-activating mutations in the HD-domain of Notch1, but not in cells that do not depend on Notch1 signaling. CJ also provoked a slight activation of NF-*κ*B, and consistent with this notion a combined treatment of CJ and the NF-*κ*B inhibitor parthenolide (Pt) led to a remarkable synergistic cell death in T-ALL cells. Altogether, our data support the concept that inhibition of the SERCA pump may be a novel strategy for the treatment of T-ALL with HD-domain-mutant Notch1 receptors and that additional treatment with the NF-*κ*B inhibitor parthenolide may have further therapeutic benefits.

T-cell acute lymphoblastic leukemia (T-ALL) is an aggressive hematologic cancer resulting from the malignant transformation of T-cell progenitors that preferentially develops in children and adolescents but is also commonly found in adults. More than 50% of human T-ALLs show auto-activation of Notch1 signaling that is responsible for the oncogenic transformation of T cells and for the impaired apoptosis and enhanced survival mechanisms of this malignancy.^1, 2, 3, 4^ Notch1 belongs to a family of conserved single transmembrane receptors that are crucial for the control of differentiation, proliferation, and apoptosis in T cells and other types of cells.^5, 6^ The canonical Notch signaling pathway is activated through a series of proteolytic cleavages (S1–S3). S1 cleavage is carried out by a Furin-like convertase in the *trans*-Golgi apparatus. Subsequently, the resulting heterodimeric receptor is transported to the membrane where it binds to Delta-Serrate-LAG or Jagged ligands from a neighboring cell. Notch1 is then cleaved by ADAM family metalloproteases (S2) and *γ*-secretase (S3) resulting in the release of the Notch1 intracellular domain (NICD), which translocates to the nucleus to promote transcription of target genes involved in cell growth.^6, 7, 8, 9^ The most frequent Notch1 mutations are observed in the heterodimerization (HD) and PEST domains.^1, 2^ Mutations of the HD domain allow a ligand-independent activation of the NICD promoting the uncontrolled growth of T-ALL cells.^10^

Although new chemotherapeutic protocols have improved the prognosis of T-ALL, the outcome of patients with resistant or relapsed leukemia remains poor,^1, 11^ highlighting the need for more effective antileukemic drugs.^9^ The identification of somatic activating Notch1 mutations in the majority of T-ALLs has stimulated interest in targeting the Notch1-signaling pathway. However, *γ*-secretase inhibitors induced only little apoptosis in human T-ALL,^12^ and exhibited strong cytotoxic effects in the gastrointestinal tract owing to inhibiting both mutant and wild-type Notch, thus limiting the use of this approach for cancer therapy as single agents.^13, 14^ By contrast, a recent report from Roti *et al.* showed that small molecule inhibitors of the sarcoendoplasmatic reticulum calcium ATPase (SERCA pump) are capable of preferentially inhibit mutant Notch1 activation, without gastrointestinal side-effects.^15^

Casearin J (CJ; Figure 1a) is a tricyclic clerodane diterpene found in *Casearia sylvestris*, a plant from the Salicaceae family. We have previously reported that representatives of this class of secondary plant metabolites are preferentially cytotoxic to leukemia cells^16^ and that they directly inhibit the SERCA pump.^17^ However, the exact mechanism of cytotoxicity and the reason for their preference toward leukemia cells remain poorly understood. Here, we addressed these issues in T-ALL cells exhibiting mutations in the HD-domain of Notch1 (CCRF-CEM), a doxorubicin-resistant version of these cells (CEM-ADR5000), and Jurkat cells that possess extracellular juxtamembrane expansion mutations of Notch1.^18^ As a consequence of SERCA pump inhibition, CJ induces calcium release from the endoplasmic reticulum (ER), promoting an increase in reactive oxygen species (ROS) and apoptosis of T-ALL cells. In addition, CJ reduces the levels of NICD and hence the mRNA levels of its target genes in T-ALL cells, which have HD-domain mutations in Notch1, but not in Jurkat cells. Furthermore, synergistic effects in cell death were observed with parthenolide (Pt), an NF-*κ*B inhibitor. Altogether, our data corroborate the notion that inhibition of the SERCA pump may be a novel strategy for the treatment of HD-domain-mutant Notch1 T-ALL and that an additional treatment with the NF-*κ*B inhibitor Pt may have further therapeutic benefits.

## Results

### CJ triggers depletion of ER Ca^2+^ pool in T-ALL cells and activates store-operated calcium entry

**Figure 1:**
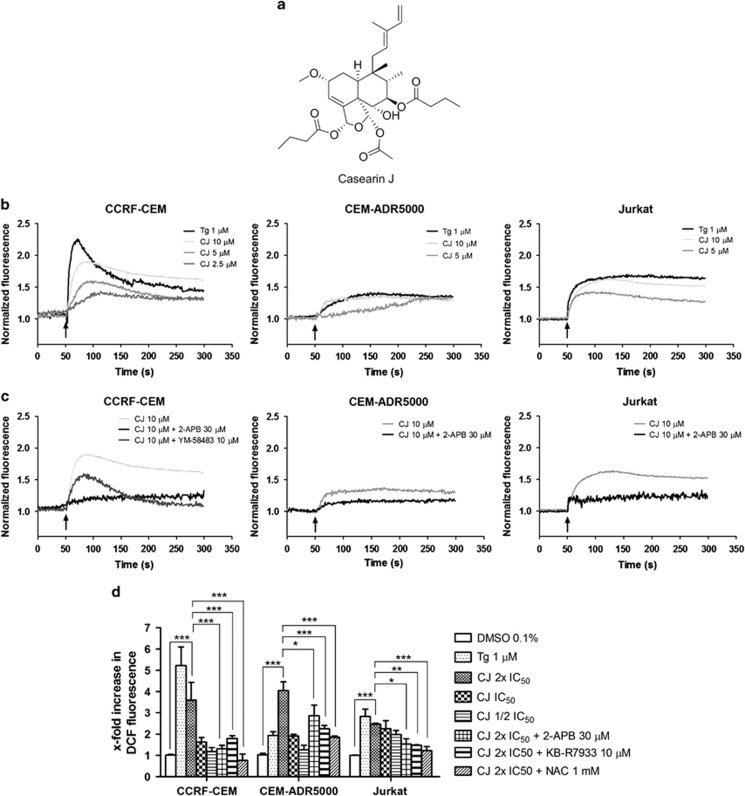
Casearin J (CJ) modulates calcium homeostasis and oxidative stress in T-ALL cells. (**a**) Structural formula of CJ. (**b**) CJ-induced instant calcium release of T-ALL cells measured with the Fluo-4 calcium probe. The calcium curves represent an average of three independent experiments. (**c**) Reduction of CJ-induced calcium release by pretreatment with an IP_3_R (2-aminophenylborate, 2-APB) or a CRAC channel inhibitor (YM-58483). The calcium curve for CJ is given again in this graph to compare the effects with the inhibitors. (**d**) Induction of oxidative stress in T-ALL cells after 24 h of incubation with CJ quantified by DCF fluorescence. As concentrations, the respective IC_50_ values of CJ were used (CCRF-CEM cells: 0.7 *μ*M; CEM-ADR5000 cells: 2.3 *μ*M; Jurkat cells: 2.5 *μ*M). The data are presented as mean±S.D. (*n*=3) and statistically analyzed by two-way ANOVA and Bonferroni's *post hoc* test. *P<0.05; **P<0.01; ***P<0.001

To determine the effect of CJ-induced SERCA pump inhibition on the ER-calcium depletion, we examined intracellular Ca^2+^ levels ([Ca^2+^]_c_) of T-ALL cells. Addition of CJ to CCRF-CEM cells in the presence of extracellular Ca^2+^ provoked a rapid, concentration-dependent biphasic increase in [Ca^2+^]_c_ reaching a Ca^2+^ peak and then descending to a sustained plateau above basal levels (Figure 1b). The same was observed in Jurkat cells. Thapsigargin (Tg), a known potent SERCA pump inhibitor, elicited a similar Ca^2+^ response but with a higher Ca^2+^ release at 1 *μ*M. On the contrary, CEM-ADR5000 cells, which are resistant to doxorubicin, released less calcium upon stimulation with Tg or CJ. This result is in line with reports showing that chemotherapy-resistant cancer cells can overexpress P-glycoprotein and possess a dysregulated calcium homeostasis, promoting resistance to SERCA pump inhibitors.^19, 20^

To confirm that inositol 1,4,5-triphosphate receptors (IP_3_Rs) were the source of the increase in [Ca^2+^]_c_, we pretreated the cells with 30 *μ*M 2-APB, an IP_3_R inhibitor. 2-aminoethoxydiphenyl borate (2-APB) pretreatment considerably prevented the increase of [Ca^2+^]_c_ (Figure 1c), suggesting that CJ induced ER Ca^2+^ pool depletion through these channels. It is known that exhaustion of the intracellular Ca^2+^ stores provokes a Ca^2+^ entry mechanism through the calcium release-activated calcium (CRAC) channel, so-called store-operated calcium entry.^21^ We therefore pretreated CCRF-CEM cells with 10 *μ*M of the CRAC channel inhibitor YM-58483. As shown in Figure 1c, this inhibitor provoked a smaller Ca^2+^ transient and prevented the induced Ca^2+^ influx through CRAC channels, returning the [Ca^2+^]_c_ back to basal levels. Altogether, these data demonstrate that CJ is able to modulate the calcium homeostasis of T-ALL cells.

### CJ-induced deregulation of calcium homeostasis promotes oxidative stress

Emptying of the intracellular Ca^2+^ stores results in a Ca^2+^ overload in mitochondria and hyperoxidation of the ER, which then causes cellular oxidative stress.^22, 23^ We therefore measured the generation of ROS by the conversion of DCFH_2_-DA to fluorescent dichlorofluorescein (DCF) after a 24 h incubation with CJ. Tg was used as a positive control for calcium-induced ROS. As shown in Figure 1d, in all three cell lines, CJ induced a concentration-dependent and statistically significant increase in DCF fluorescence, which was completely reversible by pretreatment with *N*-acetylcysteine (NAC). Notably, an increase in ROS production was also observed with CJ but not with Tg in CEM-ADR5000 cells, although this cell line shows resistance to SERCA pump inhibitors, indicating another mechanism independent of ER Ca^2+^ release, in these chemoresistant cells.

Previous studies have shown that mitochondria can buffer excess calcium released from IP_3_R through the mitochondrial calcium uniporter (mCU).^24, 25^ To study the impact of the IP_3_R and mCU on ROS production, T-ALL cells were pretreated with 2-APB or with the mCU inhibitor KB-R7943.^26^ CCRF-CEM and Jurkat cells treated with 2-APB showed a complete inhibition of CJ-induced ROS, whereas CEM-ADR5000 only exhibited a small but significant decrease. In addition, KB-R7943 was able to substantially decrease the ROS levels in all the three cell lines tested (Figure 1d), indicating that calcium influx into the mitochondria has a major role in the production of oxidative stress in T-ALL cells. To investigate whether CJ directly induces ROS production through inhibition of the respiratory chain, isolated beef heart mitochondria were treated with CJ. No inhibition was observed up to a concentration of 100 *μ*M of CJ (data not shown), suggesting that calcium release from the ER, and not a direct action of CJ on mitochondria is responsible for the induction of the ROS production.

### CJ prompts cell death of T-ALL cells by disrupting intracellular Ca^2+^ homeostasis

**Figure 2:**
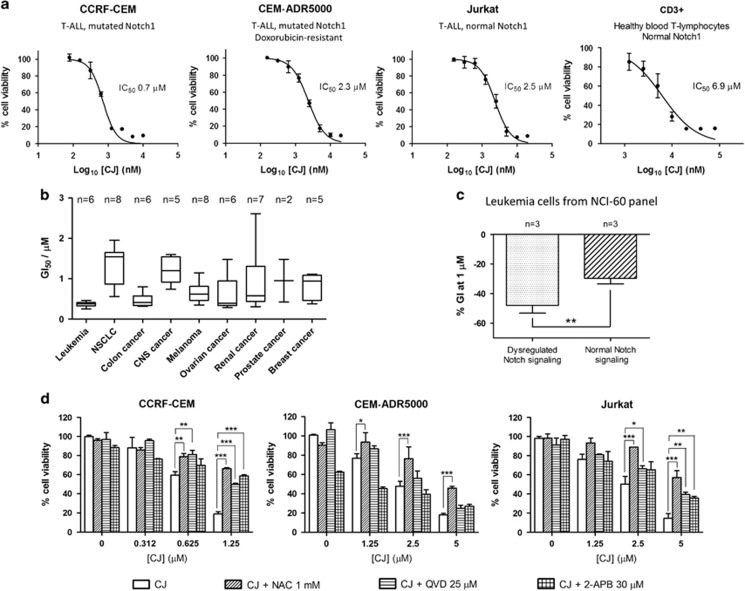
Effects of CJ on cell viability of T-ALL and CD3+ cells. (**a**) Cell viability curves were obtained with the MTT assay after 24 h of incubation. The IC_50_ value of CJ in CCRF-CEM cells was previously obtained.^17^ The data are presented as mean±S.D. (*n*= 3), the IC_50_ values were calculated by non-linear regression. (**b**) Growth inhibition (GI) of CJ tested with the sulphorodamine assay in the NCI-60 panel of human cancer cell lines. (**c**) Comparison of the GI of CJ at 1 *μ*M in cells with dysregulated Notch signaling (CCRF-CEM, MOLT-4, HL-60) *versus* cells with normal Notch (K-562, RPMI-8226, SR), **P<0.01. (**d**) Characterization of the mechanism of cytotoxicity using the reactive oxygen species (ROS) scavenger *N*-acetylcysteine (NAC), the IP_3_R blocker 2-APB, and the caspase inhibitor QVD-OPh. Concentrations indicate the respective IC_50_ values of CJ (see figure legend 1). Data are expressed as mean±S.D. (*n*=3) and statistically analyzed by two-way ANOVA and Bonferroni's *post hoc* test. *P<0.05; **P<0.01; ***P<0.001

Next, we determined whether SERCA pump inhibition by CJ had any effect on cell survival. We previously reported potent cytotoxic effects of CJ in CCRF-CEM cells (IC_50_=0.7 *μ*M).^17^ Continuing these studies, we now assessed the viability of other T-ALL cells (CEM-ADR5000 and Jurkat cells) as well as CD3+ cells from healthy human donors after 24 h of CJ treatment using the 3-(4,5-dimethylthiazol-2-yl)-2,5-diphenyltetrazolium bromide (MTT) assay. As shown in Figure 2a and b, CJ was able to induce cell death in T-ALL cells in the low micromolar concentration range. CCRF-CEM cells were more susceptible to cell death than CEM-ADR5000 and Jurkat cells (Figure 2a). Although CJ was also cytotoxic to normal CD3+ cells isolated from human blood, T-ALL cells were significantly more susceptible to SERCA pump inhibition when comparing IC_50_ values (*P*<0.01). For example, although 1.25 *μ*M CJ reduced the cell viability of CCRF-CEM cells by 82%, the same concentration reduced cell survival of CD3+ cells by 14% (Figure 2a). In addition, CJ was tested in the NCI-60 panel of cell lines showing an overall preferential cytotoxicity to leukemia cells (Figure 2b). Interestingly, CJ promoted strong growth inhibition (GI) at 1 *μ*M in leukemia cells with dysregulated Notch signaling, whereas this effect was significantly less in cells with normal Notch alleles (Figure 2c).

To study the impact of Ca^2+^ release from the ER on cell viability, we pretreated the T-ALL cells with the IP_3_R inhibitor 2-APB. In agreement with the results from our calcium release assay, inhibition of IP_3_R prevented CJ-induced cell death in CCRF-CEM and to a lesser extent in Jurkat cells but not in CEM-ADR5000 (Figure 2d). Also NAC was able to protect the cells from CJ-induced cell death, suggesting that oxidative stress was a mediator of cell death induced by calcium release in T-ALL cells. To determine whether the reduced cell viability was due to apoptosis cells were pretreated with QVD-OPh, a pancaspase inhibitor,^27^ before CJ was added and cell death measured by MTT. QVD-OPh was able to partially restore the cell viability in CCRF-CEM and Jurkat cells, suggesting that apoptosis is involved in cell death. Surprisingly, the doxorubicin-resistant CEM-ADR5000 cells showed only a slight effect with QVD-OPh. This is in line with reports that caspase inhibitors promote alternative cell death pathways and that drug-resistant cells are more prone to necrosis upon cytotoxic drugs when caspases are inhibited.^28, 29^

### CJ induces the intrinsic mitochondrial signaling pathway in T-ALL

**Figure 3:**
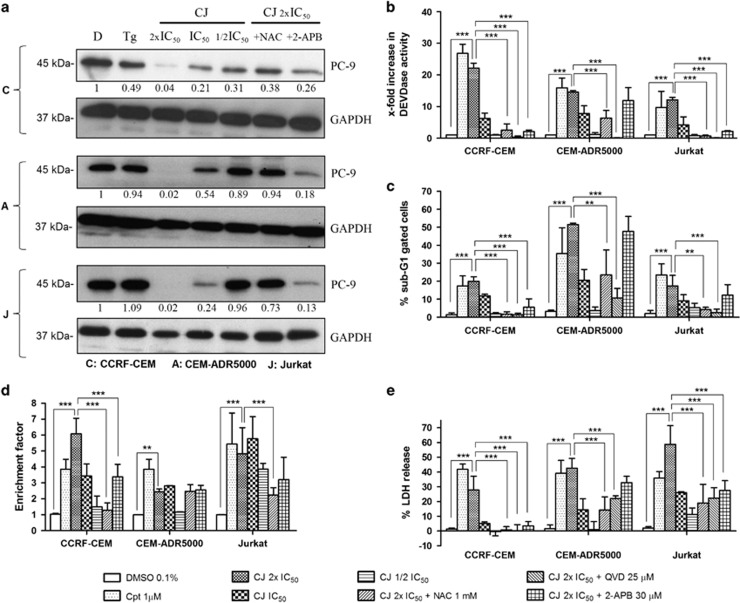
CJ induces apoptosis in T-ALL cells. (**a**) Proteolytic processing/activation of procaspase-9 (PC-9) after 24 h of incubation (*n*=2) as shown by the disappearance of the inactive pro-form of caspase-9. A representative immunoblot is shown. Quantification of the immunoblots was performed with Image J taking the ratio of PC-9/GAPDH of the negative control as 1. NAC: 1 mM. 2-APB: 30 *μ*M. Tg: 1 *μ*M. (**b**) Caspase-3/-7 activation measured as DEVDase enzymatic activity. Cpt: camptothecin. (**c**) Quantification of the sub-G1 population of T-ALL cells stained with propidium iodide as a measure of DNA degradation by FACS. (**d**) Histone-associated DNA fragments detected by ELISA. (**e**) Membrane integrity assay for the detection of lactate dehydrogenase release to the extracellular space. Concentrations indicate the respective IC_50_ values of CJ (see figure legend 1). All experiments were carried out after 24 h of incubation with CJ. The data are presented as mean±S.D. (*n*=3) and statistically analyzed by two-way ANOVA and Bonferroni's *post hoc* test. **P<0.01; ***P<0.001

Treatment of cells with cytotoxic drugs can initiate intrinsic apoptosis. We therefore measured the activation of procaspase-9 (the apical caspase of the intrinsic apoptotic pathway) and its target, effector caspases-3/-7. T-ALL cells treated with CJ showed a concentration-dependent processing/activation of procaspase-9 that is reversed by blocking oxidative stress (Figure 3a) in all three cells lines, but only partially prevented by inhibiting the IP_3_Rs. CCRF-CEM cells showed the best response to the IP_3_R inhibitor, as shown by the quantification of the immunoblots, correlating with the previous results. Similarly, CJ caused a dose-dependent activation of caspase-3/-7, which was markedly inhibited by 2-APB, NAC and QVD-OPh in CCRF-CEM, and Jurkat cells. Again no effect was observed with 2-APB in CEM-ADR5000 (Figure 3b). Caspase-3/-7 activation leads to DNA degradation, one of the hallmarks of apoptosis. Therefore, fluorescence-activated cell sorting (FACS) analyses were carried out to quantify the increase in sub-G1 DNA-containing cells. In accordance with the caspase-3/-7 assay, CJ-treated T-ALL cells showed increased levels of hypodiploid DNA (sub-G1) in a concentration-dependent manner. This increase was prevented by pretreating CCRF-CEM with QVD-OPh, NAC, or 2-APB (Figure 3c). Jurkat and CEM-ADR5000 cells showed a different behavior although 2-APB was not able to restore the CJ-induced sub-G1 population in these cell lines as much as in CCRF-CEM cells.

We wanted to confirm the sub-G1 data by measuring cytoplasmic histone-associated DNA fragmentation using the Cell Death Detection ELISA kit (Roche). In the three cell lines tested CJ was able to induce DNA degradation (Figure 3d). CJ-induced degradation of CCRF-CEM cells was largely diminished, albeit not totally prevented by NAC and 2-APB. To determine whether CJ-treated cells also underwent secondary apoptosis or necrosis we quantified the release of lactate dehydrogenase (LDH) from cells with damaged cell membranes. A dose-dependent increase in LDH was noted in all three cell lines (Figure 3e). Interestingly, the strongest LDH release was seen in Jurkat and CEM-ADR5000 and much less in the CCRF-CEM. On the other hand, 2-APB, NAC, and QVD primarily prevented LDH release in CCRF-CEM cell line, but only partially in Jurkat and CEM-ADR5000 cells. These results indicate that apart from apoptosis, CJ also induces uncontrolled necrosis, but with different impact from calcium release or oxidative stress in different cell lines. Altogether, our data demonstrate that CJ-induced SERCA pump inhibition, followed by IP_3_R-mediated Ca^2+^ release and oxidative stress induces apoptotic cell death in CCRF-CEM cells as well as other cell death mechanisms in the resistant CEM-ADR5000 and Jurkat cells.

### Inhibition of the SERCA pump by CJ influences mutant Notch1 signaling

**Figure 4:**
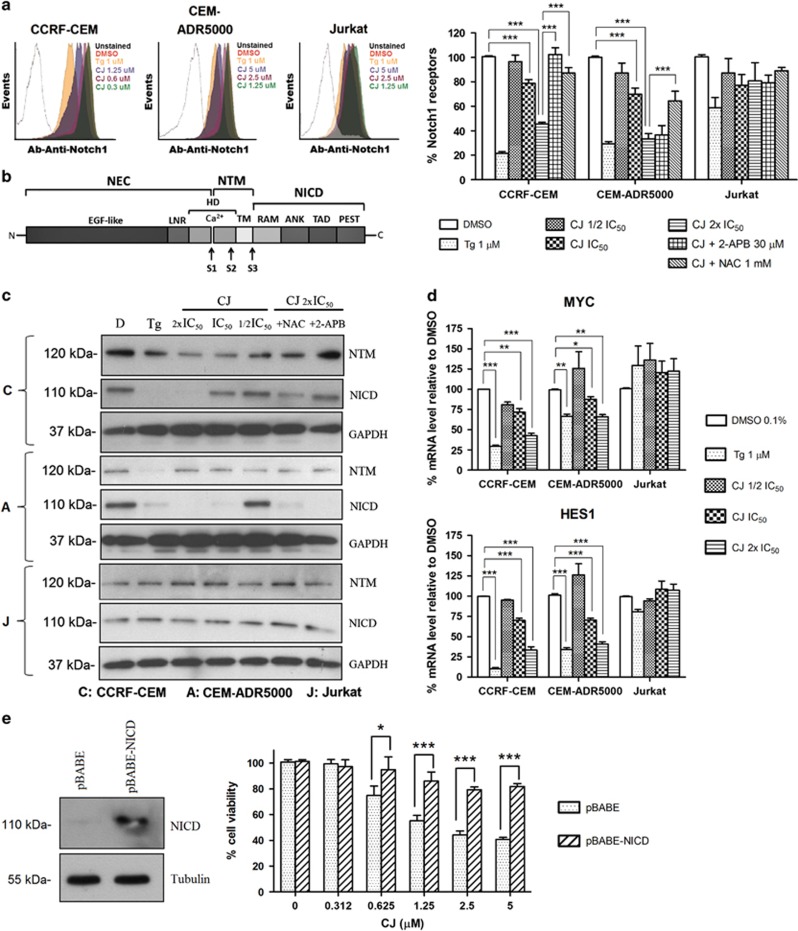
CJ preferentially influences mutant Notch1 signaling in T-ALL cells. (**a**) Cell surface expression of Notch1 receptors quantified by FACS analysis after treatment of the cells with CJ alone or after pretreatment with the IP_3_R inhibitor 2-APB or NAC; a representative experiment is shown on the left panel. (**b**) Schematic assembly of the Notch1 receptor. (**c**) Immunoblotting of Notch1 transmembrane region (NTM) and Notch1 intracellular domain (NICD) after treatment of the cells with CJ alone or after pretreatment with 2-APB or NAC. A representative immunoblot is shown. (**d**) qRT-PCR to determine the mRNA expression level of the Notch1 target genes *MYC* and *HES1*. Concentrations indicate the respective IC_50_ values of CJ (see Figure legend 1). (**e**) Immunoblot of NICD in pBABE-NICD-transduced CCRF-CEM cells (left panel, *n*=2; a representative blot is shown) and cell viability experiments of the empty vector and the NICD-transduced cells treated with CJ (right panel). All experiments were carried out after 24 h of incubation with CJ. Data are presented as mean±S.D. (*n*=3) and statistically analyzed by two-way ANOVA and Bonferroni's *post hoc* test. *P<0.05; **P<0.01; ***P<0.001

The apparent preference of CJ toward T-ALL cells with dysregulated Notch1 signaling prompted us to study how CJ may influence Notch1. We first determined whether Notch1 was still transported to the membrane surface after stimulation of the cells with CJ. As shown in Figure 4a, the surface expression of Notch1 was significantly reduced in CCRF-CEM and CEM-ADR5000 cells in a concentration-dependent manner with only small changes in Jurkat cells (Figure 4a). Moreover, inhibition of ER-calcium release through the IP_3_Rs completely restored surface expression of Notch1 after stimulation with CJ in CCRF-CEM cells, but not in CEM-ADR5000 cells. Notably, in the presence of NAC, Notch1 was also maintained on the cell surface of CCRF-CEM and CEM-ADR5000 (Figure 4a). These data suggest that inhibition of SERCA pump with subsequent release of calcium and ROS production leads to a preferential inhibition of Notch exocytosis and signaling when it is mutated in the HD-domain.

Notch1 is processed by furin, which is a vital step in the maturation process and occurs at the S1 cleavage site in the Golgi apparatus (Figure 4b).^1^ To analyze the impact of CJ on this maturation step, protein extracts of CJ-treated T-ALL cell lines were immunoblotted to detect the Notch1 furin-processed transmembrane subunit (NTM, 120 kDa). CJ reduced the levels of NTM in a concentration-dependent manner in CCRF-CEM cells, whereas no effect was observed in CEM-ADR5000 or Jurkat cells (Figure 4c). Interestingly, only 2-APB was able to recover the expression of NTM, indicating that calcium release is responsible for this effect.

Moreover, HD-domain-mutant Notch1 is auto-activated once it reached the membrane in two cleavage steps (S2 and S3) (Figure 4b). To study if reduced expression of Notch1 at the surface correlates with lower levels of NICD, immunoblotting was performed. As expected, NICD levels were reduced after CJ treatment in a concentration-dependent manner (Figure 4c) and this was again prevented by 2-APB or NAC (Figure 4c), being in line with the FACS results. NICD levels were also strongly reduced by CJ in CEM-ADR5000 cells, but in contrast to CCRF-CEM cells, the inhibitors were not able to recover the basal levels of NICD, suggesting a different mechanism of inhibition. Jurkat cells showed no decrease of NICD or NTM levels after SERCA pump inhibition (Figure 4c).

Once NICD is released from the membrane, it translocates to the nucleus and activates its target genes (e.g., *MYC*, *HES1*). We therefore performed quantitative real-time PCR of a series of NICD target genes after 6 h of CJ treatment. Low micromolar concentrations of CJ significantly reduced the expression of *MYC* and *HES1* in CCRF-CEM and CEM-ADR5000 cells. On the contrary, no effect was observed in Jurkat cells (Figure 4d), supporting the immunoblotting and FACS results.

To further clarify the role of Notch1 in the mechanism of cytotoxicity of CJ in CCRF-CEM cells, NICD was overexpressed by transducing cells with an empty pBABE vector or with pBABE-NICD (Figure 4e, left panel). Cell viability studies demonstrated that CCRF-CEM cells are protected from CJ-induced cell death when a non-inhibitable NICD is overexpressed. This effect is absent with the empty pBABE vector (Figure 4e, right panel).

### CJ synergizes with the NF-*κ*B inhibitor Pt

**Figure 5:**
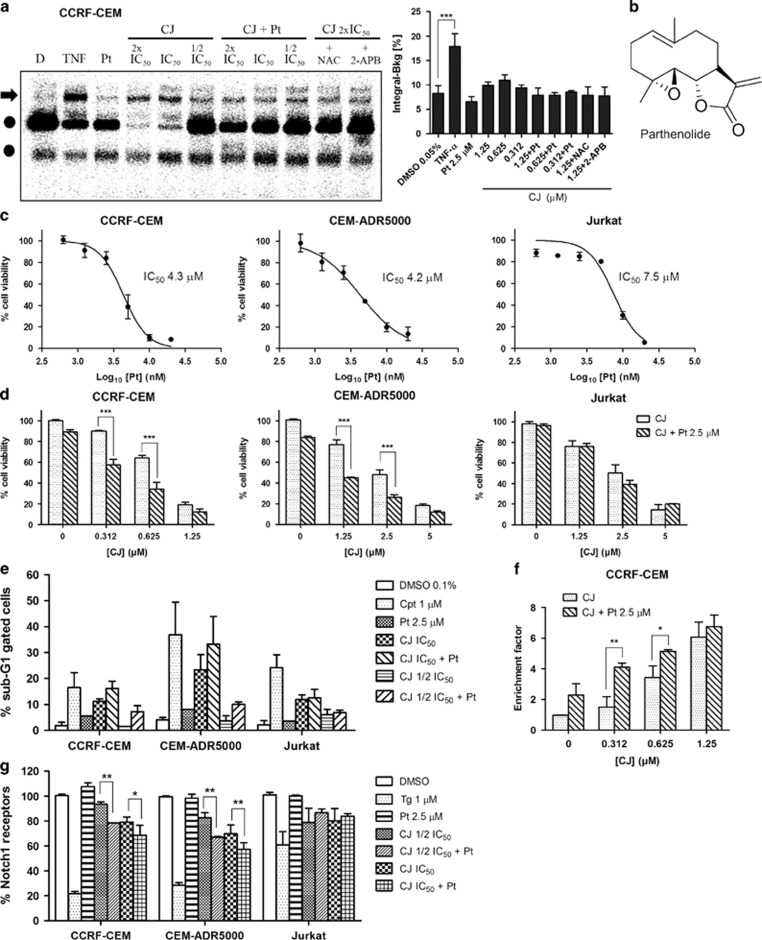
Synergistic inhibitory effects of CJ in combination with parthenolide (Pt). Cells were pretreated for 1 h with Pt and then with different concentrations of CJ for 24 h. Concentrations of CJ indicate its IC_50_ value in CCRF-CEM cells (0.7 *μ*M). (**a**) EMSA showing NF-*κ*B DNA binding after treating CCRF-CEM cells with CJ alone or together with Pt (2.5 *μ*M). TNF: tumor necrosis factor-α (4 ng/ml). NAC (1 mM). 2-APB (30 *μ*M). The arrowhead indicates the position of NF-*κ*B DNA complexes. The circle denotes a non-specific activity binding to the probe. One representative EMSA is shown. (**b**) Structural formula of Pt. (**c**) Cell viability curves of T-ALL cells with Pt after 24 h of incubation using the MTT assay. (**d**) Cell viability of the three cell lines after single and dual treatment of CJ and Pt using the MTT assay. (**e**) Quantification of the sub-G1 population of T-ALL cells stained with propidium iodide as a measure of DNA degradation by FACS analysis after single and combined treatment. Cpt: camptothecin. (**f**) Histone-associated DNA fragments detected by ELISA. (**g**) Notch1 cell surface expression in T-ALL cells after single and dual treatment. Data are presented as mean±S.D. (*n*= 3) and statistically analyzed by one-way ANOVA (**a**) or two-way ANOVA (**d**–**g**) and Bonferroni's *post hoc* test. *P<0.05; **P<0.01; ***P<0.001

NF-*κ*B activation by ER stress requires both an increase in intracellular levels of Ca^2+^ and ROS.^30^ CJ caused the emptying of the ER-calcium stores and oxidative stress in T-ALL cells. Therefore, we studied whether treatment of cells with CJ had an impact on the activation of NF-*κ*B. Electrophoretic mobility shift assay (EMSA) analysis showed a slight increase in NF-*κ*B DNA binding in CJ-treated CCRF-CEM cells (Figure 5a). These observations prompted us to evaluate whether T-ALL cells were more susceptible to a dual inhibition of the SERCA pump and NF-*κ*B. Targeting the NF-*κ*B pathway in Notch1 mutant T-ALL cells has already been proposed,^4, 15^ but the combination of an NF-*κ*B and a SERCA pump inhibitor has not been reported so far. We therefore used Pt (Figure 5b), a natural compound, known to inhibit NF-*κ*B and to readily induce apoptosis in cancer cells.^31, 32^

First, the IC_50_ values of Pt in the three cell lines were determined (Figure 5c). Subsequently, the three cell lines were pretreated for 1 h with a low cytotoxic concentration of Pt (2.5 *μ*M) and increasing concentrations of CJ for 24 h. Pt alone had no effect on NF-*κ*B DNA binding but it prevented the CJ-induced NF-*κ*B activation (Figure 5a). Moreover, CJ-induced NF-*κ*B DNA binding was slightly reduced by pretreating the cells with NAC and 2-APB (Figure 5a). Interestingly, the combined treatment caused a rapid decline in the cell viability of CCRF-CEM and CEM-ADR5000 cells as shown by the MTT assay (Figure 5d). The synergistic effect was observed at the lowest tested concentrations of CJ except for Jurkat cells. Accordingly, similar synergistic effects were also observed in the apoptosis assays (Figure 5e and f). To get further insights in the synergistic effects, cell surface expression of Notch1 was studied under the dual treatment of T-ALL cells. As shown in Figure 5g, Pt alone is unable to modulate the expression of Notch1 receptors at the surface of the membrane, but the combination resulted in a slight, but statistical significant decrease as compared with CJ alone, an effect that may contribute to the synergistic effect of apoptosis induction in Notch1 mutant cells.

## Discussion

Membrane proteins, such as Notch receptors, are folded and processed in the ER/Golgi apparatus before being displayed on the cell surface.^6, 33, 34^ Maturation of Notch receptors requires the proteolytic cleavage by a furin-like protease at the S1 cleavage site. A non-covalently bonded heterodimer is formed consisting of the ligand-binding extracellular domain (NEC) and the transmembrane domain (NTM; Figure 4b). Calcium is required for the proper folding and the structural integrity of this heterodimer.^35, 36, 37^ The ER is the most important intracellular Ca^2+^ store.^38^ Regulation of intracellular Ca^2+^ by the ER is mainly mediated by the SERCA pump, which shuttles Ca^2+^ from the cytosol into the ER. Therefore, SERCA pump inhibitors, such as the sesquiterpene lactone Tg, have been described to intervene in the maturation process of Notch1 receptors and to be strong inductors of apoptosis in HD-domain-mutant T-ALL cells.^15^

We here demonstrate that natural tricyclic clerodane diterpenes, such as CJ, can also affect the Notch1-signaling pathway in human T-ALL cells. We show for the first time that CJ is able to induce ER-calcium depletion through the IP_3_R as a direct consequence of SERCA pump inhibition. This effect in turn provokes mitochondria to buffer the excess cytosolic calcium through mCU and to induce the generation of mitochondrial ROS. Mitochondrial matrix Ca^2+^ overload triggers cytochrome c release, caspase-9, and -3 activation, and leads to apoptosis.^39, 40^ Our results revealed that CJ-induced apoptosis, measured as caspase-9 and caspase-3/-7 activation and DNA degradation, was more pronounced in non-resistant Notch1 HD-mutant cells than in leukemia cells with extracellular juxtamembrane mutations in Notch1. Apoptosis was dependent on calcium release from the IP_3_Rs and oxidative stress, corroborating a pivotal role of these two responses in the mechanism of action of this natural product. Doxorubicin-resistant T-ALL cells exhibited a certain resistance to SERCA pump inhibition, and therefore calcium release through IP_3_Rs did not have a major role in the mechanism of CJ-induced apoptosis. In contrast, Jurkat cells seemed to exert a different mechanism of cell death. Besides of apoptosis, necrosis was also observed in this cell line, suggesting that in the absence of Notch1 mutations in the HD-domain CJ may induce other effects, which are not related to this transcription factor.

Altogether, our results indicate that Notch1 has an important role in the cytotoxic mechanism of CJ as the compound reduced the furin processing of Notch1, cell surface expression of Notch1 receptors, and prevented the formation of the cleaved NICD-signaling molecule. These effects were preferentially visible in T-ALL cells with HD-domain mutations of Notch1, but not in cells that do not depend on Notch1 signaling. In addition, overexpression of NICD protects CCRF-CEM cells from CJ-induced cell death. This is in line with studies from Roti *et al.*^15^ They have shown that SERCA pump inhibition by Tg preferentially prevents the maturation of mutant Notch1, whereas normal receptors are still expressed. In addition, we provide evidence that the increase in oxidative stress may also contribute to this effect. High amounts of calcium-induced ROS prevented the expression of the receptors on the cell surface and consequently the auto-activation of NICD. Owing to ER-calcium release, unfolded and misfolded proteins, such as mutant Notch1, could accumulate in the ER lumen leading to the activation of the unfolded protein response (UPR), ROS production, and apoptosis.^34, 41^ The interplay between misfolded proteins in the ER, oxidative stress, activation of UPR, and apoptosis has recently been reported.^42^ This scenario could explain the observed cell death induced by CJ and why the IP_3_R receptor inhibitor 2-APB and the antioxidant NAC were so effective in blocking this cell death in T-ALL cells.

**Figure 6:**
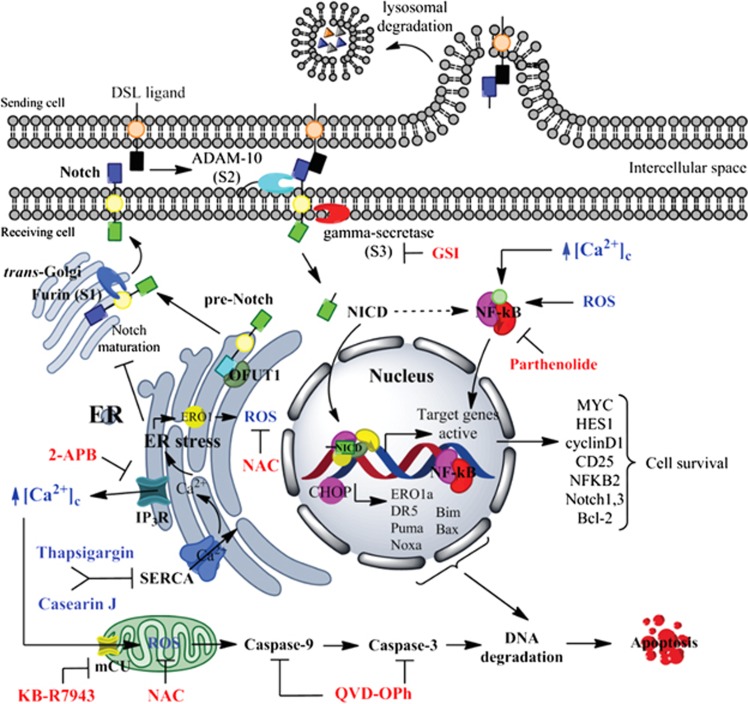
Notch 1 signaling pathway and proposed mechanism of action of CJ. Notch is synthesized in the ER as pre-Notch. After the maturation process, Notch1 is transported by vesicle trafficking to the plasma membrane where it is activated, releasing NICD. HD-domain mutations induce a ligand-independent activation of Notch1. NICD translocates to the nucleus to promote transcription of target genes such as *MYC*, *HES1*, *IL2R*, *NFKB2*, *BCL-2*, and *NOTCH1* itself. NICD also activates NF-*κ*B. Thus, modulation of the cell cycle, differentiation, and inhibition of apoptosis takes place in a cooperative mechanism when Notch1 and NF-*κ*B are involved in T-ALL. CJ-induced SERCA pump inhibition induces calcium release through the IP_3_R, which is buffered by mitochondria through the mCU channel, inducing a mitochondrial calcium overload followed by an induction of oxidative stress. This ER stress impairs signaling of the oncogenic mutant Notch1 and activates the intrinsic apoptotic pathway and finally cell death of T-ALL cells. Notably, a combination of SERCA pump and NF-*κ*B inhibition shows synergistic effects in cell death

NICD, the maturation product derived from Notch1, is a transcription factor regulating the expression of genes, such as *MYC* or *HES1*. Myc is an oncogenic transcription factor that controls cell growth and metabolism. Therefore, the aberrant cell proliferation and increased survival of T-ALL are partly controlled by the Notch1-Myc transcriptional network.^43^ The Notch1-Hes1 regulatory axis is implicated in the upregulation of PI3K and NF-*κ*B signaling as well as in the inhibition of apoptosis by repressing the proapoptotic BH3-only protein Puma.^44^ We could demonstrate that CJ was also able to downregulate the Notch1 target genes *MYC* and *HES1*, which may contribute to the induction of apoptosis. Taken together, our results indicate that the CJ-induced repression of mutant Notch1 re-activates the apoptotic machinery in T-ALL cells and strengthen the rationale for the use of SERCA inhibitors in the treatment of Notch1 mutant T-ALL, and probably of those cancers with mutant Notch1. This proposed mechanism is depicted in Figure 6.

Constitutively active Notch1 activates NF-*κ*B by directly stimulating the IKK signalosome.^45, 46^ Inhibition of this pathway can efficiently prevent tumor growth *in vitro* and *in vivo*.^4, 47^ Accordingly, the combination of CJ and the NF-*κ*B inhibitor Pt synergistically reduced cell viability and enhanced apoptosis in HD-domain-mutant T-ALL cells, either resistant to doxorubicin or not. In agreement with Vilimas *et al.*,^4^ we were unable to observe synergistic effects in Jurkat cells, which are known to be more resistant to NF-*κ*B inhibition^4^ and insensitive to γ-secretase inhibitors treatment.^5^ The combination of Pt and CJ had a stronger impact on the cell surface expression of HD-domain-mutant Notch1 receptors than the single compounds alone, indicating the involvement of auto-activated Notch1 in the synergism. Summarizing our results and considering the well-known function of NF-*κ*B in the control of cell survival, we propose that a dual inhibition of SERCA pump and NF-*κ*B could be a novel promising approach to treat T-ALL in the future. However, further work is needed to validate the combined therapy in *in vivo* tumor models.

## Materials and Methods

### Cell lines and reagents

CCRF-CEM and CEM-ADR5000 cells were obtained as a gift from Professor T Efferth, Department of Pharmaceutical Biology, Johannes Gutenberg University, Mainz, Germany. Jurkat cells were obtained from ATCC (clone E6-1, ATCC TIB-152). All T-ALL cell lines were cultured in RPMI 1640 (Invitrogen, Carlsbad, CA, USA) supplemented with 10% heat-inactivated fetal bovine serum, penicillin (100 U/ml), and streptomycin (100 *μ*g/ml) at 37 °C under 5% CO_2_. Antibodies to procaspase-9 (catalog #9502), NTM (#4380) and NICD (catalog #2421 and #4147) were from New England Biolabs (Frankfurt, Germany), and to GAPDH from Merck-Millipore (Darmstadt, Germany) (MAB374, clone 6C5). For flow cytometry, antibodies to Notch1 receptor and isotype control were obtained from BD Bioscience (San Jose, CA, USA) (clone MHN-519 and P3.6.2.8.1, respectively). The following inhibitors were used: 2-APB, YM-58483, KB-R7943, and Pt from Sigma-Aldrich (Darmstadt, Germany), NAC from Roth, and QVD-OPh from MP Biomedicals (Heidelberg, Germany).

### Isolation of CD3+ cells from human blood

CD3+ cells were isolated by using human CD3 microbeads (MACS, Miltenyi Biotec, Bergisch Gladbach, Germany) according to the manufacturer's protocol. In brief, PBMCs were first isolated from human buffy coats of healthy donors (ethical permission number from the ethics commission, University of Freiburg: 356/13; 2013) by using a density-gradient medium (LymphoPrep). Isolated PBMCs were immediately incubated with CD3 microbeads for 15 min at 4 °C and separated using a magnetic column. CD3+ cells were obtained and immediately used after separation. RPMI medium was used for the incubation of the cells.

### Calcium release

Cytosolic calcium in T-ALL cells was monitored using a Fluo-4 direct calcium assay kit (Thermo Fisher Scientific, Langenseibold, Germany) according to the manufacturer's protocol. 1.5 × 10^5^ cells were seeded in 96-well plates and incubated with Fluo-4-AM for 30 min at 37 °C and 5% CO_2_ and subsequently 30 min at room temperature. After incubation, the cells were washed with PBS and 100 *μ*l of assay buffer were added to each well. The [Ca^2+^]_c_ was monitored in real-time using a Varioskan Flash multimode reader (Thermo Fisher Scientific) for a total of 300 s. A baseline of 50 s was measured prior to addition of CJ. Tg was used as positive control for ER-calcium depletion. Cells were pretreated with inhibitor molecules 1 h prior to treatment with CJ.

### Measurement of intracellular ROS formation

The dye 2',7'-dichlorofluorescin diacetate (DCFH-DA), which is oxidized to the DCF by hydroperoxides, was used to measure relative levels of cellular peroxides as previously described.^48^ In brief, 2 × 10^6^ T-ALL cells were seeded into six-well plates and incubated with different concentrations of CJ for 24 h. In case of the use of an inhibitor, cells were stimulated 1 h with the inhibitor prior to treatment with CJ. The cells were centrifuged and washed with PBS and incubated with RPMI medium containing 20 *μ*M DCFH-DA for 30 min in the dark. Subsequently, cells were washed with PBS, resuspended in RPMI medium and the fluorescence was measured at an excitation wavelength of 485 nm and an emission wavelength of 530 nm using a microplate fluorescence reader. Protein quantification by Bradford assay was done to normalize the values.

### Measurement of ROS in bovine heart mitochondria

Superoxide dismutates rapidly to H_2_O_2_. Thus, the total rate of superoxide and H_2_O_2_ production was measured by the horseradish peroxidase-dependent oxidation of Amplex Red.^49^ The 1 ml assay contained 50 *μ*g bovine heart mitochondrial membranes, 2 U horseradish peroxidase and 10 *μ*M Amplex Red. The reaction was started by the addition of 30 *μ*M NADH in the presence of 10 *μ*M piericidin. When indicated, 1-100 *μ*M CJ were added. The oxidation of the nucleotide was followed at 340 nm; the production of resorufin was monitored at 557 minus 620 nm (*ɛ*=23.1/mM/cm at pH 6.0) using an UV/vis diode array photometer (J&M; Aalen, Germany).

### Cell viability

MTT assay was carried out for quantitative analysis of cell viability as previously described.^16^ In brief, cells were seeded in 96-well plates and incubated with CJ for 24 h at 37 °C and 5% CO_2_. In case of the use of an inhibitor, cells were stimulated 1 h with the inhibitor prior to treatment with CJ. MTT solution (5 mg/ml) was added to each well after the incubation period, and the absorbance was measured at 595 nm after 2 h incubation by dissolving the formazan crystals in DMSO. IC_50_ values were calculated by non-linear regression using GraphPad Prism.

### Tumor cell line profiling in the NCI-60 panel

Tumor cell line profiling was performed by the National Cancer Institute (NCI) as part of the NCI-60 Human Tumor Cell line Anticancer Drug Screen (http://dtp.nci.nih.gov). GI potential of CJ was determined with the sulphorodamine assay in five different concentrations.

### Caspase-3/7 activity

Cells were seeded in six-well plates at a density of 2 × 10^6^ cells/well and incubated for 24 h with CJ at various concentrations. After the incubation, cells were washed with PBS and resuspended in lysis buffer (31 mM Hepes-KOH pH 7.4, 2.5 mM EGTA, 2.5 mM MgCl_2_, 12 mM DTT, 120 *μ*M PMSF, 12 ng/ml leupeptin, 500 ng/ml pepstatin, 12 *μ*g/ml aprotinin, 1.5 *μ*g/ml cytochalasin B). Three freeze–thaw cycles were carried out by freezing in liquid nitrogen and thawing in a 40 °C water bath with subsequent vortexing. Protein extracts were centrifuged at 20 800 × *g* and 4 °C for 10 min and the concentration was quanitifed using the BSA kit (BioRad, München, Germany). The fluorescence emitted by the release of 7-amino-4-methylcumarin (AMC) from the caspase-3/-7 substrate (Ac-DEVD-AMC) was monitored in a Fluostar Optima plate reader with an excitation wavelength of 370 nm and an emission wavelength of 450 nm. Relative fluorescence unit (RFU) values were calculated via the ratio of average rate of the fluorescence increase and protein concentration. RFU sample values were referred to negative controls (untreated cells) and given as fold increase values.

### Cell-cycle measurement by FACS

In total, 1 × 10^6^ cells were seeded in 1 ml of culture medium in 12-well plates, incubated overnight, and exposed to CJ for 24 h. After exposure, cells were pelleted, washed with PBS, and fixed in 2 ml of ice cold 70% ethanol and kept at 4 °C overnight. Afterwards, cells were centrifuged at 1500 rpm for 10 min and resuspended in PBS containing 0.1 mg/ml RNase and 0.25 mg/ml propidium iodide. The cells were incubated for 30 min at 37 °C and 5% CO_2_ and the DNA content of cells was measured by a FACScalibur (BD Biosciences). In total, 10  000 gated events were analyzed for each sample.

### Cell death detection ELISA

Determination of cytoplasmic histone-associated DNA fragments was determined spectrophotometrically at 405 nm using the Cell Death Detection ELISA kit (Roche Diagnostics, Mannheim, Germany) according to the manufacturer's protocol. In brief, 1 × 10^6^ cells were seeded in six-well plates and incubated with CJ for 24 h. After the incubation period, the ELISA was carried out. The enrichment factor was calculated by comparing the absorbance units with the negative control.

### LDH-release assay

LDH-release assay was carried out for the quantitative determination of cytotoxicity owing to cell membrane permeabilization using the Cytotoxicity Detection kit (LDH) (Roche Diagnostics) according to the manufacturer's protocol. In brief, cells were seeded in six-well plates at a density of 2 × 10^6^ cells/well in RPMI 1640 culture medium. After incubation, cells were treated with CJ for 24 h. The absorbance was measured at 490 nm. Total LDH release (100%) was obtained by the treatment of cells with 2% Triton-X. The relative LDH release is defined by the ratio of LDH released over total LDH in the intact cells.

### Notch1 cell surface staining

In total, 1 × 10^6^ cells were seeded into 12-well plates and incubated for 24 h with CJ. Cells were harvested and washed twice with a PBS buffer containing 0.5% bovine serum albumin and 0.02% sodium azide. Each sample was treated separately with either Notch1 antibody or isotype control antibody for 1 h. After the incubation period the cells were washed twice with the washing buffer and subjected to FACS analysis using the FL4 channel.

### Immunoblotting

In total, 2 × 10^6^ cells were seeded into six-well plates in 2 ml of culture medium and incubated for 24 h. After stimulation, cells were pelleted, washed with PBS, resuspended in lysis buffer (HEPES 20 mM pH 7.9, NaCl 350 mM, glycerol 20%, NP-40 1%, MgCl_2_ 2.5 mM, EDTA 0.5 mM, EGTA 2.5 mM, DTT 12 mM, PMSF 125 *μ*M, Aprotinin 12 *μ*g/ml, Pepstatin 500 ng/ml, 12 *μ*g/ml aprotinin), and incubated at 4 °C for 30 min. Cell lysates containing equal amount of protein were separated by SDS-PAGE, transferred to PVDF membranes and immunoblotted with antibody against procaspase-9, NICD, or GAPDH. Immunoreactive bands were detected with an ECL advanced chemiluminescence detection reagent (Amersham Pharmacia Biotech, Pittsburgh, PA, USA). Immunoblots were quantified using Image J (NIH).

### RNA isolation, cDNA synthesis, and qRT-PCR

Total RNA from T-ALL cells was isolated using the RNeasy Plus Mini Kit from Qiagen (Hilden, Germany) and converted to single-strand cDNA using the QuantiTect Reverse Transcription Kit from Qiagen according to the manufacturer's protocol. The analysis of mRNA expression profiles was performed with qRT-PCR. Primers and probes were designed with Oligo Architect online v4.0 from Sigma-Aldrich. The data were analyzed using the ΔΔCT method and plotted as percentage of transcript compared with negative control. In a 25 *μ*l PCR reaction, 2 *μ*l of cDNA (corresponding to 20 ng of total RNA input) was amplified in a LightCycler 480 using 2 × conc. LightCycler 480 Probes Master, 50 nM primers and 100 nM probe for the 18 S rRNA reference gene (fwd: 5′-CGGCTACCACATCCAAGG-3′, rev: 5′-CGGGTCGGGAGTGGGT-3′, probe: 5′-[HEX]-TTGCGCGCCTGCTGCCT-[TAM]-3′) or 300 nM primers and 100 nM probe for the gene of interest. The following target gene primers and probes were used: human MYC fwd: 5′-CATGGTGAACCAGAGTTTC-3′, rev: 5′-CAGTCCTGGATGATGATG-3′, probe: 5′-[6-FAM]-CGGACGACGAGACCTTCATCA-[TAM]-3′. human HES1 fwd: 5′-GCTGATAACAGCGGAATC-3′, rev: 5′-TGTGGCTACTTGGTGATC-3′, probe: 5′-[6-FAM]-TCTCCTTGGTCCTGGAACAGC-[TAM]-3′.

### Cloning of human NICD

EF.hNICD.CMV.GFP was a gift from Linzhao Cheng (Addgene plasmid #17623) to clone human NICD into the retroviral expression vector pBabe.puro. NICD was amplified by PCR to create *BamHI* (NICD-*BamHI*-fwd: 5′-ATATGGATCCGCCACCATGCGGCGGCAGCATG-3′) and *EcoRI* (NICD-*EcoRI*-rev: 5′-GAGAGAATTCTTACTTGAACGCCTCCGGG-3′) restriction sites for subcloning in the pBABE vector.

### Retroviral infection of NICD

Viral particles to infect CCRF-CEM cells were produced in HEK 293 T cells. In total, 2 × 10^6^ cells were seeded in 10-cm plates. On the day of transfection, 3 *μ*g of the plasmid pBABE.puro.hNICD or empty vector (pBABE), 3 *μ*g enveloping vector VSVg, and 3 *μ*g packaging vector Hit60 were adjusted to 300 ml in serum-/antibiotics free Opti-MEM Life Technologies (Darmstadt, Germany) and gently mixed with 30 *μ*l attractene transfection reagent (Qiagen). The transfection mix was added to the cells for 6 h. Cells were then incubated for 8 h in DMEM supplemented with 10% FCS, 1% Pen/Strep and 5 mM sodium butyrate (Sigma, Taufkirchen, Germany). The virus were harvested from the supernatant, 5 *μ*g/ml polybrene (Sigma) was added to increase infection efficiency. Fresh supernatant was then used for infection of 1 × 105 CCRF-CEM cells. Cells were resuspended in 400 *μ*l virus supernatant. For spinfection, cells were plated in 12-well plates and centrifuged at 2000 rpm for 10 min. After centrifugation, infected cells were incubated for 1 h at 37 °C before supplementation with 1 ml full medium. Transfected cells and cells with empty vector were used for the MTT assay as described above.

### EMSA NF-*κ*B

In total, 2 × 10^6^ cells were seeded into six-well plates and incubated for 24 h with CJ. Cells stimulated with human TNF-α (4 ng/ml) for 30 min served as a positive control for NF-*κ*B activation. After stimulation with CJ, cells were pelleted, washed with PBS, and lysed in totex lysis buffer. In case of the use of an inhibitor cells were stimulated 1 h with the inhibitor prior to treatment with CJ. The Bradford protein assay (BioRad) was used to determine the concentration of the protein extract. The EMSA was performed as previously described by Könczöl *et al.*^50^

### Statistical analysis

Data are expressed as means±S.D. One-way ANOVA or two-way ANOVA with Bonferroni's *post hoc* test was used for the statistical significances depending on the setting of the experiment. Results were considered significant with *P*<0.05, *P*<0.01 very significant, and *P*<0.001 as highly significant. Three independent experiments were carried out with all the above-mentioned methods unless otherwise stated.

## Abbreviations

T-ALL: T-cell acute lymphoblastic leukemia
CJ: casearin J
SERCA: sarcoendoplasmic reticulum calcium ATPase
NICD: Notch1 intracellular domain
NTM: Notch1 transmembrane region
HD-domain: heterodimerization domain
IP_3_Rs: inositol 1,4,5-triphosphate receptors
2-APB: 2-aminoethoxydiphenyl borate
NAC: *N*-acetylcysteine
Pt: parthenolide
Tg: thapsigargin
